# Sally and… Cozmo? Training theory of mind using a toy robot: a pilot study

**DOI:** 10.3389/fpsyg.2025.1620345

**Published:** 2025-12-01

**Authors:** Davide Ghiglino, Federica Floris, Davide De Tommaso, Agnieszka Wykowska

**Affiliations:** 1 Social Cognition in Human-Robot Interaction, Istituto Italiano di Tecnologia, Genova, Italy; 2 Piccolo Cottolengo Genovese di Don Orione, Genova, Italy; 3 SIDiN, Società Italiana Disturbi del Neurosviluppo, Firenze, Italy

**Keywords:** autism spectrum disorder, robot-assisted therapy, social cognition, theory of mind, commercial robotics

## Abstract

**Introduction:**

Children with autism spectrum disorder (ASD) often show impairments in Theory of Mind (ToM). This pilot study examined whether a commercially available toy robot (Cozmo) could support false-belief training within an Applied Behavior Analysis (ABA) framework.

**Methods:**

Fourteen children with ASD completed a two-period crossover design comparing robot-assisted training with standard therapy. Sessions used adapted false-belief tasks (Deceptive Box, Sally-Anne) delivered through therapist-controlled interactions with Cozmo. ToM and emotion recognition were assessed with NEPSY-II at T0, T1, and T2.

**Results:**

Robot-assisted sessions produced greater improvements than standard therapy alone on both NEPSY-II Emotion Recognition and ToM subscales, as confirmed by mixed-effects models.

**Discussion:**

Findings suggest that an affordable toy-like robot can be feasibly integrated into structured clinical interventions and may enhance ToM-related outcomes in children with ASD, warranting further evaluation in larger samples.

## Introduction

Autism spectrum disorders (ASD) are neurodevelopmental conditions characterized by persistent difficulties in social communication and interaction, often accompanied by restricted and repetitive patterns of behavior (American Psychiatric Association [APA], 2013). Among the core cognitive challenges observed in individuals with ASD, deficits in Theory of Mind (ToM; Premack and Woodruff, 1978)—the ability to attribute mental states such as beliefs, desires, and emotions to oneself and others—are particularly significant (Baron-Cohen et al., 1985). ToM impairments can lead to difficulties in predicting and understanding social behavior, ultimately impacting social adaptation and quality of life (Schulte et al., 2022).

Traditional interventions to foster ToM in children with ASD include role-play, storytelling, or structured exercises facilitated by therapists (Wellman et al., 2001). While these methods can improve performance on specific tasks, their generalization to everyday contexts is often limited, especially for children who struggle with spontaneous social interaction (Terlouw et al., 2021). Moreover, conventional interventions are highly dependent on continuous therapist mediation, which can reduce scalability and accessibility for families already facing significant financial and time burdens.

In recent years, socially assistive robots (SARs) have been proposed as promising tools to complement existing interventions. Robots can deliver simplified, consistent, and contingent cues, thereby scaffolding children’s social and cognitive learning within their zone of proximal development (Vygotsky, 1980; Bruner, 1975). Studies have shown that robots can enhance children’s engagement and motivation in therapeutic contexts (Scassellati et al., 2012; Kouroupa et al., 2022). Unlike static materials such as dolls or pictures, embodied and interactive robots afford perception–action coupling and contingent feedback, two features known to enhance engagement and learning in children with ASD. However, systematic reviews emphasize that while robots can effectively support short-term engagement, evidence for generalization to naturalistic social settings remains limited, and further controlled studies are needed (Begum et al., 2016; Diehl et al., 2012).

Most research in this field has relied on humanoid robots such as Nao, Kaspar, or iCub, which can imitate human gestures and model joint attention or imitation (Robins et al., 2009; Wainer et al., 2014; Santos et al., 2023). These platforms can successfully elicit social responses, but their high cost and technical complexity make them less feasible for widespread use outside research labs or specialized clinics (Scassellati et al., 2018). In contrast, non-humanoid or toy-like robots, though less sophisticated, offer advantages in terms of affordability, portability, and ease of integration into therapeutic routines (Dickstein-Fischer et al., 2018; Sandygulova et al., 2022). Their playful appearance and exaggerated animations may reduce intimidation and sustain attention, making them particularly suitable for children (Anzalone et al., 2015). At the same time, the contribution of such robots requires empirical validation, since their reduced behavioral repertoire might limit the kinds of social interactions they can scaffold. The present work focuses on Theory of Mind training using classical developmental psychology paradigms. False-belief tasks such as the Sally–Anne and Deceptive Box remain among the most widely used assessments of ToM in both typical and atypical development (Baron-Cohen et al., 1985; Hogrefe et al., 1986; see also Korkiakangas et al., 2016). Despite their prominence, these tasks have rarely been adapted as training tools. We chose to build on these well-established paradigms precisely because they are immediately recognizable to clinicians and easy to integrate into existing therapeutic routines, without the need for extensive additional training. Although false-belief tasks were originally developed for assessment rather than training, their structured nature and clear outcome measures make them suitable for repeated practice when embedded within a scaffolded framework. In this view, systematic variation of distractors, temporal delays, and prompt-fading allows children to rehearse perspective-taking in increasingly demanding contexts, strengthening the underlying ability to attribute and decouple mental states. Embedding them in an interactive robotic framework allowed us to examine whether technologies unavailable to earlier researchers can transform classical tasks from static assessments into motivating, scaffolded practice opportunities. Therefore, we tested the feasibility and short-term effectiveness of using a commercially available toy robot, Cozmo, to deliver structured false-belief training in children with ASD. The intervention was implemented within an ABA-based therapeutic framework and conducted in a clinical setting under therapist supervision. While the intervention was conducted within a clinical environment under therapist supervision, our objective was to examine the feasibility and potential benefits of repurposing a commercial toy robot for structured ToM exercises. If effective, such an approach could later inform home-based educational interventions implemented by caregivers. Therefore, our aim was to investigate whether embedding familiar ToM paradigms in robot-assisted activities could enhance children’s ToM and emotion recognition skills beyond standard therapy. We hypothesized that the interactive and engaging nature of robot-mediated training would lead to measurable improvements, providing a first step toward evaluating the translational potential of affordable robotic platforms in ASD interventions.

## Materials and methods

### Participants

For each child taking part in the study, written informed consent signed by their parents/legal caregivers was collected after the information about the design, the procedure, and the rationale of the project was provided. Fourteen children took part in the training with the Cozmo robot (Age = 12.07 ± 2.13, 3 females; mean IQ = 84.79 ± 17.45). All participants had a formal diagnosis of autism spectrum disorder confirmed by standardized assessments [Autism Diagnostic Observation Schedule (ADOS) and Autism Diagnostic Interview–Revised (ADI-R)]. Diagnostic evaluations were first carried out by the local public health service and subsequently confirmed at the Boggiano Pico clinical center, ensuring consistency of classification. In addition to IQ, participants’ functional level was determined according to the DSM-5 severity criteria. All children were classified as Level 1 (“requiring support”), which indicates that they were verbal and able to participate in structured tasks with guidance. This restriction was intentional: given the pilot nature of the study and the complexity of false-belief reasoning, we opted to recruit children with the highest likelihood of completing the training.

Due to ethical constraints, raw ADOS and ADI-R scores were not accessible. Likewise, systematic data on comorbidities or specific verbal IQ measures were not available at this stage. These limitations are acknowledged in the Discussion, where we highlight the importance of including a broader range of participant characteristics in future studies to account for inter-individual variability.

Our experimental protocols followed the ethical standards laid down in the Declaration of Helsinki and were approved by the local Ethics Committee (Comitato Etico Regione Liguria).

### Training design

**FIGURE 1:**
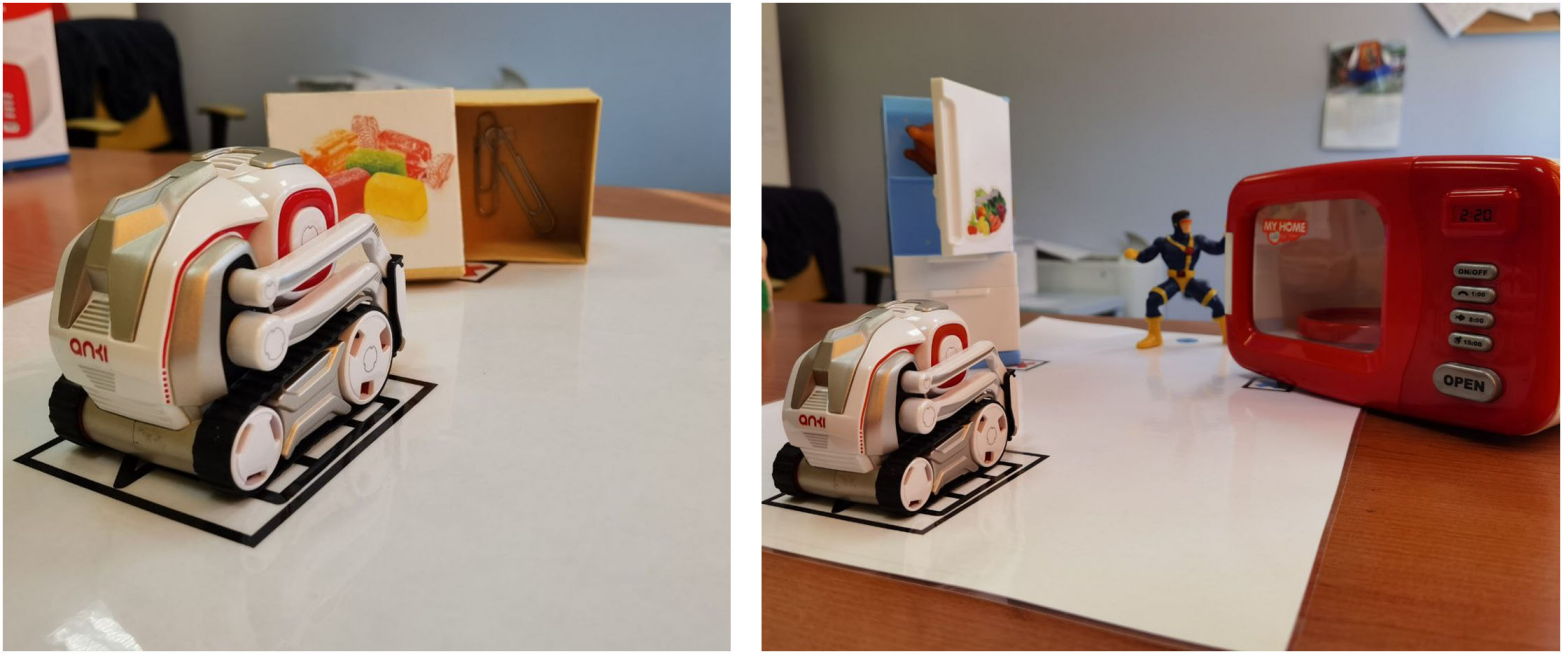
Examples of the false-belief scenarios used in the robot-assisted intervention. On the left: adaptation of the Deceptive Box Task. On the right: adaptation of the Sally-Anne Task.

The training program incorporated the toy robot Cozmo (Anki Robotics, San Francisco, CA, United States) into an Applied Behavior Analysis (ABA)-based therapeutic framework to assess its potential for enhancing false-belief understanding and emotion recognition in children with ASD. Cozmo (Figure 1) is a compact, commercially available robot primarily white and gray with red accents, equipped with treaded wheels, a small mechanical arm, and a digital display used to convey animated “face” expressions through a proprietary “emotion engine.” Its design, developed by animators with experience at Pixar, was deliberately inspired by the aesthetics of friendly cartoon characters, giving Cozmo an appearance that children often find approachable and engaging. The robot combines basic mobility with a repertoire of pre-programmed vocalizations and gestures that can be triggered via a tablet interface, while its ability to display distinct emotions makes interactions feel lively and responsive. We selected Cozmo because it offers affordability and portability together with sufficient social expressiveness to sustain children’s engagement, making it a practical complement to therapist-led interventions. Despite its toy-like form, the robot’s repertoire of pre-programmed gestures, vocalizations, and emotional displays is sufficient to sustain attention and support role-play activities.

Cozmo was operated via a tablet-based application under Wizard-of-Oz control, meaning that the therapist triggered the robot’s actions (animations, gaze shifts, vocalizations, and feedback) at predetermined points in the task. The therapist was always in charge of the session: they monitored the child’s behavior, determined when to deliver prompts or reinforcement, and adjusted the level of support if attention began to fade. In both the robot-assisted and standard therapy conditions, therapists used a consistent prompting hierarchy, moving from verbal to gestural and finally to physical prompts if necessary, in line with ABA practice.

Each session consisted of two tasks: an adaptation of the Deceptive Box Task (Hogrefe et al., 1986) and the Sally–Anne Task (Baron-Cohen et al., 1985). The Deceptive Box Task involved presenting a familiar container (e.g., a candy box) with an unexpected content inside, requiring the child to infer what an uninformed observer (Cozmo) would believe about its contents. In the Sally–Anne Task, Cozmo played the role of Sally, while the child acted as Anne. The robot placed an object in a designated location before “falling asleep” (i.e., deactivating momentarily). During this period, the child, guided by the therapist, moved the object to a new location. Upon “waking up,” Cozmo prompted the child to predict where it would look for the object, assessing their ability to attribute and differentiate mental states.

The training followed an incremental complexity structure across sessions. Initially, tasks were presented with minimal distractors and direct prompts. As the training progressed, the difficulty increased by introducing additional visual and spatial distractors, such as miniature household toys (e.g., a fridge or a microwave) that served as alternative hiding places. These additions required children to track multiple potential locations and coordinate them with the robot’s perspective. Difficulty was further increased by extending the temporal delay between hiding and query, and by systematically fading prompts as the child demonstrated competence. Advancement to the next level required a child to achieve at least 50% accuracy in the current session. If a child struggled, they were allowed up to two repeated attempts on different days before moving forward.

A two-period crossover design was implemented to compare the effectiveness of robot-assisted training versus standard therapy alone. Participants were randomly assigned to two groups: one received robot-assisted training first, followed by standard therapy, while the other received standard therapy first before undergoing robot-assisted training. This design ensured that all participants experienced both conditions, minimizing individual variability and allowing within-subject comparisons. To evaluate the intervention’s impact, children’s performance was assessed at three time points: before the intervention (T0), after the first training period (T1), and after the second training period (T2), with pre-to-post changes analyzed using NEPSY-II ToM and Emotion Recognition subscales.

### Data analysis

To mitigate the effect of confounding factors due to the heterogeneity of ASD manifestation the dependent variable of the analyses related to the improvement on each NEPSY-II subscale was the difference (Δ) calculated between two subsequent assessments (T1−T0; T2−T1). For data analysis, a series of mixed-effect models were applied to investigate the overall effect of the robot-assisted training (treated as the fixed factor) net of potential individual differences (treated as the random factor), regardless of the order of administration of the treatment. Analyses were conducted using the lme4 package (Bates et al., 2003) in RStudio Team (2015). Parameter estimates (β) and their associated *t*-tests (t, p), were calculated using the Satterthwaite approximation for degrees of freedom (Kuznetsova et al., 2017) and presented to show the magnitude of the effects, with bootstrapped 95 % confidence intervals (Efron and Tibshirani, 1994).

## Results

**FIGURE 2:**
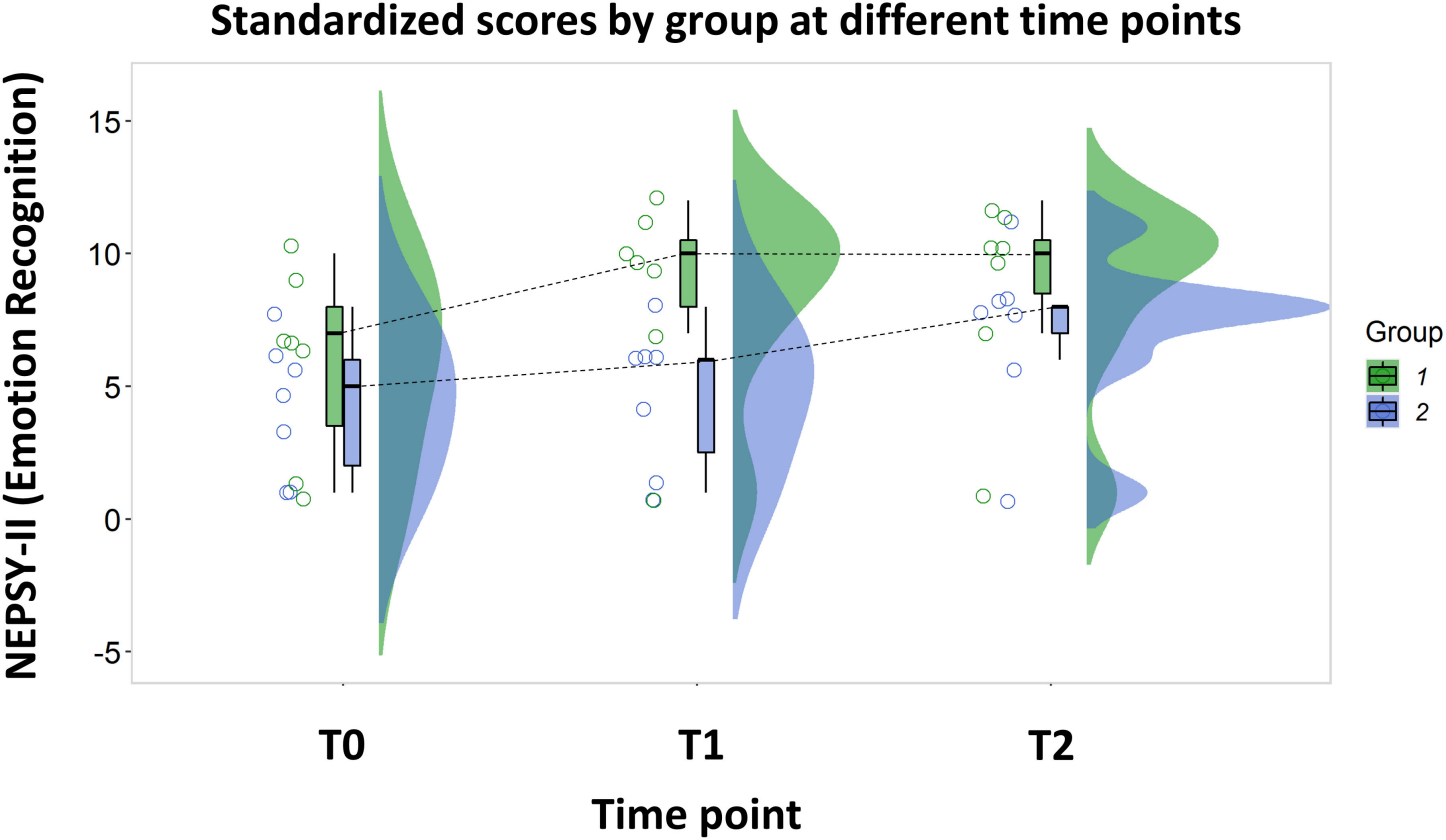
Rain-cloud plot representing the standardized scores of the Emotion Recognition subscale of NEPSY-II at different time points. Group 1 underwent the activities with the robot during the period between T0 and T1, Group 2 underwent the same activities in the period between T2 and T1.

We found a significant difference between the treatments on children’s Emotion Recognition abilities [β_Estimate_ = 2.43 (CI_Lower_ = 1.37, CI_Upper_ = 3.48), t_13_ = 4.66, *p* < 0.001]. Namely, combining the activities with the robot with the standard therapy improved children abilities more than when the standard therapy was applied alone (Average Δ_Combined_ = 2.64, SE Δ_Combined_ = 0.41; Average Δ_Standard_ = 0.21, SE Δ_Standard_ = 0.41; see Figure 2 for the detailed comparison between time points).

**FIGURE 3:**
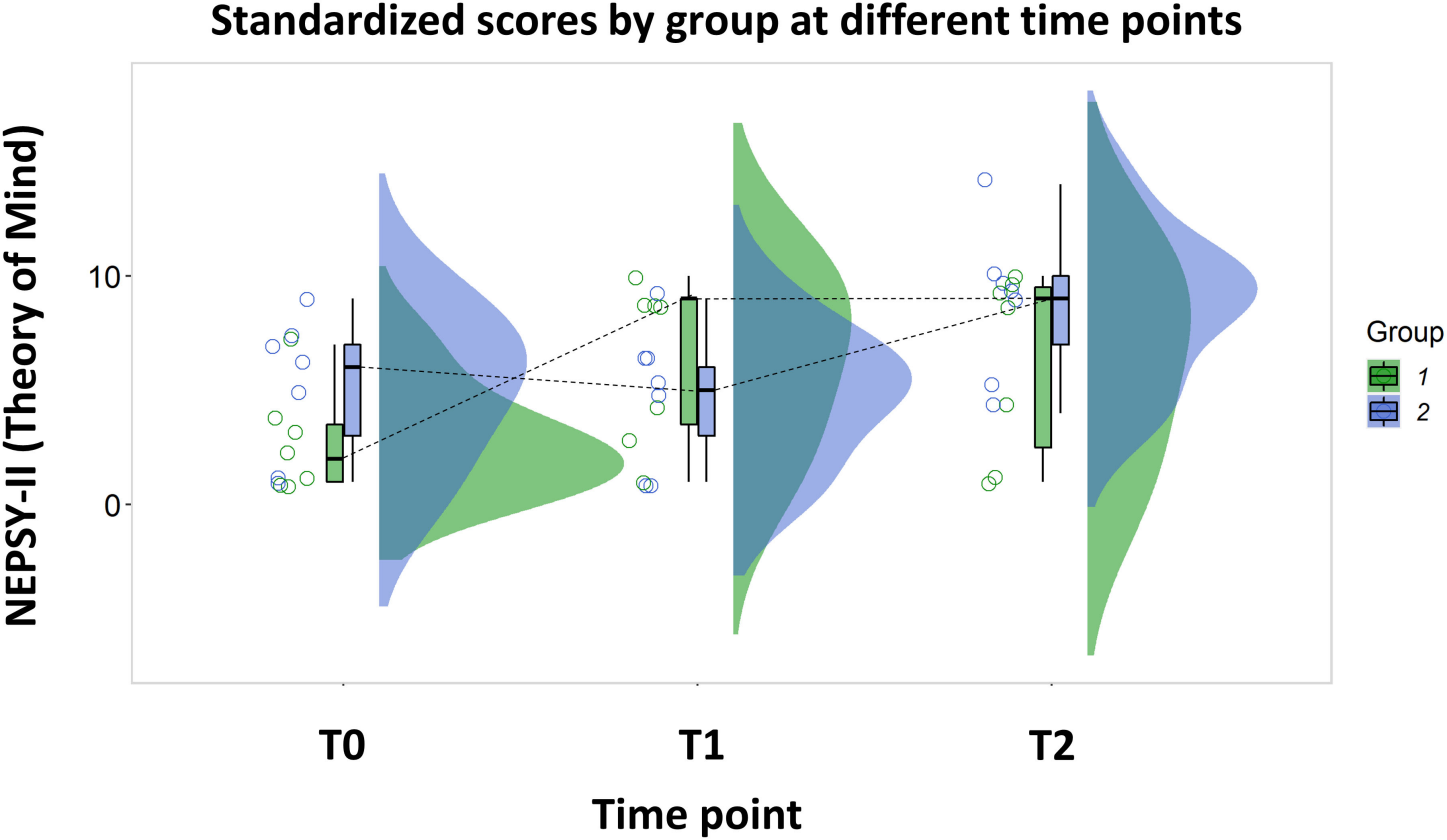
Rain-cloud plot representing the standardized scores of the Theory of Mind subscale of NEPSY II at different time points. Group 1 underwent the activities with the robot during the period between T0 and T1, Group 2 underwent the same activities in the period between T2 and T1.

The same pattern was found when we analyzed Theory of Mind abilities [β_Estimate_ = 4.14 (CI_Lower_ = 2.84, CI_Upper_ = 5.45), t_13_ = 6.42, *p* < 0.001]. The combination between the activity with the robot and the standard therapy was more effective than the standard therapy alone in improving children’s ToM abilities (Average Δ_Combined_ = 3.86, SE Δ_Combined_ = 0.49; Average Δ_Standard_ = −0.29, SE Δ_Standard_ = 0.49; see Figure 3 for the detailed comparison between time points).

Therefore, for both NEPSY-II subscales, the activity with the robot improved the efficacy of standard therapy.

## Discussion

The findings of this pilot study provide preliminary evidence supporting the use of commercial toy robots as viable tools for Theory of Mind (ToM) training in children with autism spectrum disorder (ASD). Our results indicate that embedding robot-assisted activities within an Applied Behavior Analysis (ABA)-based therapeutic framework enhances social cognition skills, particularly false-belief understanding and emotion recognition. These findings align with previous research demonstrating the potential of social robotics in ASD intervention (Scassellati et al., 2012; Kouroupa et al., 2022), while also extending this knowledge by focusing on the feasibility of a commercially available, non-specialized robot.

A key contribution of this study is the demonstration that a toy-like, non-humanoid robot can be effectively integrated into ToM training. Classic false-belief paradigms, such as the Deceptive Box and Sally–Anne tasks, have traditionally been administered with dolls, everyday objects, or picture-based materials (Baron-Cohen et al., 1985; Hogrefe et al., 1986). In this context, the distinctive contribution of the robot lies not in the novelty of the task itself, but in the way the task is experienced by the child. Unlike static materials, robots provide contingent feedback, consistent timing, and a dynamic presence that sustains engagement and motivates participation. Whereas humanoid robots such as Nao or Kaspar offer rich motor repertoires and human-like gestures (Robins et al., 2009; Wainer et al., 2014), their cost and technical complexity limit accessibility. By contrast, Cozmo represents an affordable and portable alternative. Although its behavioral repertoire is limited, its cartoon-like design and expressive animations, inspired by Pixar/Disney characters, make it approachable and engaging, highlighting the potential role of toy-like robots as complementary clinical tools.

A distinctive aspect of our design is the use of false-belief tasks as training material. These paradigms remain the gold standard in developmental psychology for assessing ToM (Wellman et al., 2001). We chose to embed them in the training precisely because they are familiar to clinicians and easy to integrate into existing routines. Importantly, assessment of ToM and emotion recognition was carried out independently using the NEPSY-II subscales, not with the same false-belief tasks used in training, thereby minimizing carry-over effects due to repeated exposure. From a developmental perspective, the repetition of false-belief paradigms can be considered structured practice rather than simple retesting: by varying distractors, introducing temporal delays, and fading prompts, the intervention provided scaffolded opportunities for children to rehearse perspective-taking in increasingly demanding contexts. This approach is consistent with developmental theories emphasizing the importance of graduated support in acquiring complex cognitive skills (Vygotsky, 1978; Bruner, 1983; Ghiglino et al., 2021, 2023).

Our results also highlight the role of engagement and motivation in sustaining children’s participation. Previous research has shown that autistic children often struggle to maintain attention in social skills training (Sandygulova et al., 2022). The interactive and responsive behaviors of Cozmo appeared to capture and sustain engagement throughout the sessions. The robot’s expressive feedback likely functioned as a form of reinforcement, consistent with principles of ABA, and may have contributed to the observed improvements (Dickstein-Fischer et al., 2018).

Although the intervention was designed with the potential for home use in mind, the present study was carried out in a clinical setting under therapist supervision. The robot functioned as a tool under the therapist’s control, complementing ongoing interventions rather than acting autonomously. Thus, the results demonstrate feasibility and short-term efficacy in a structured clinical environment, but they do not yet confirm effectiveness in everyday domestic contexts. Furthermore, despite these promising findings, several limitations should be acknowledged. First, the sample size was small, which limits generalizability. Second, all participants were classified as DSM-5 Level 1 (“requiring support”), ensuring that they were verbal and able to participate in structured tasks, but restricting applicability to children with greater support needs. The inclusion criteria—limited to children at DSM-5 functional Level 1—mean that results cannot be extrapolated to children with higher support needs. Third, for ethical reasons we could not access raw ADOS and ADI-R scores, nor did we systematically collect measures of verbal IQ or comorbidities, which may have contributed to variability in outcomes. Indeed, while most children demonstrated improvements, some showed minimal gains, underscoring the need to consider individual characteristics in tailoring interventions. Future studies should therefore address these limitations by recruiting larger and more heterogeneous samples, incorporating a broader range of clinical and cognitive measures, and extending the duration of training to examine longitudinal effects. Importantly, subsequent research should also expand beyond false-belief paradigms to encompass a wider variety of social scenarios, such as intention understanding, deception, and cooperative problem-solving. These directions will be crucial to determine whether the benefits of robot-assisted ToM training generalize beyond task-specific gains to broader aspects of social functioning.

In conclusion, this pilot study provides initial evidence for the feasibility of integrating an affordable, commercially available toy robot into structured ToM interventions for children with ASD. While the study was conducted entirely in a clinical setting under therapist supervision, the findings highlight the potential of toy-like robots to complement established practices and pave the way for future work exploring their use in more naturalistic or home-based contexts. Leveraging accessible robotic platforms for scalable, cost-effective ASD interventions represents a promising direction for future research and clinical practice. Future work should also extend robot-assisted activities to a wider range of social scenarios, such as deception, intention understanding, and cooperative problem-solving, in order to foster broader generalization beyond false-belief reasoning.

## Conclusion

This pilot study provides preliminary evidence that a commercially available toy robot can be integrated into structured interventions to support Theory of Mind training in children with ASD. Embedding Cozmo within an ABA-based therapeutic framework proved feasible and engaging, and led to measurable gains in false-belief understanding and emotion recognition. The use of an affordable and portable robotic platform highlights the potential of accessible technologies to complement traditional interventions and broaden therapeutic options. While further studies with larger and more heterogeneous samples are needed, our findings point to a promising direction for developing cost-effective, scalable supports that enhance the accessibility of social-cognitive training in ASD.

## Data Availability

The raw data supporting the conclusions of this article will be made available by the authors, without undue reservation.
